# Metabolic pseudoprogression in a patient with metastatic KIT exon 11 GIST after 1 month of first-line imatinib: a case report

**DOI:** 10.3389/fonc.2023.1310452

**Published:** 2023-12-20

**Authors:** Elisa Tassinari, Nicole Conci, Giacomo Battisti, Francesco Porta, Valerio Di Scioscio, Maria Giulia Pirini, Dario de Biase, Maria Concetta Nigro, Miriam Iezza, Fausto Castagnetti, Luigi Lovato, Stefano Fanti, Maria Abbondanza Pantaleo, Margherita Nannini

**Affiliations:** ^1^ Department of Medical and Surgical Sciences (DIMEC), University of Bologna, Bologna, Italy; ^2^ Nuclear Medicine, IRCCS Azienda Ospedaliero-Universitaria di Bologna-Policlinico di Sant’Orsola, Bologna, Italy; ^3^ Department of Pediatric and Adult Cardio-Thoracovascular, Oncoematologic and Emergencies Radiology Unit, IRCCS Azienda Ospedaliero-Universitaria di Bologna, Bologna, Italy; ^4^ Pathology Unit, IRCCS Azienda Ospedaliero-Universitaria di Bologna-Policlinico di Sant’Orsola, Bologna, Italy; ^5^ Department of Pharmacy and Biotechnology (FaBit), University of Bologna, Bologna, Italy; ^6^ Hematology “Lorenzo E Ariosto Seràgnoli”, IRCCS Azienda Ospedaliero-Universitaria Di Bologna, Bologna, Italy; ^7^ Oncology Unit, IRCCS Azienda Ospedaliero-Universitaria di Bologna, Bologna, Italy

**Keywords:** gastrointestinal stromal tumors, GIST, functional imaging, FDG-PET, imatinib

## Abstract

**Background:**

Positron emission tomography (PET) with 18-fluorodeoxyglucose (^18^FDG) has proven to be highly sensitive in the early assessment of tumor response in gastrointestinal stromal tumors (GIST), especially in cases where there is doubt or when the early prediction of the response could be clinically useful for patient management. As widely known, kinase mutations have an undoubtful predictive value for sensitivity to imatinib, and the inclusion of KIT and PDGFRa mutational analysis in the diagnostic workup of all GIST is now considered standard practice.

**Case presentation:**

Herein, we described in detail a case of an exon 11 KIT mutated-metastatic GIST patient, who presented an unexpected metabolic progression at the early ^18^FDG-PET evaluation after 1 month of first-line imatinib, unconfirmed at the liver biopsy performed near after, which has conversely shown a complete pathological response.

**Conclusions:**

This report aims to highlight the existence of this metabolic pseudoprogression in GIST at the beginning of imatinib therapy in order to avoid early treatment discontinuation. Therefore, an early metabolic progression during a molecular targeted therapy always deserves to be evaluated in the context of the disease molecular profiling, and in case of a discordant finding between functional imaging and molecular background, a short-term longitudinal control should be suggested.

## 1 Background

Gastrointestinal stromal tumors (GISTs) are the most common mesenchymal tumors of the gastrointestinal tract arising from the interstitial cells of Cajal (ICCs), with an incidence of approximately 10 to 15 cases per million population per year (1).

GISTs have always been considered the milestone of precision oncology, from when KIT mutations were recognized as the main pathogenetic driver, soon becoming the GIST therapeutic target (2–5). Since then, mutational analysis of KIT and PDGFRA has assumed a proven predictive value for sensitivity to molecular targeted therapies, and its inclusion in the diagnostic workup of all GISTs has become standard practice (6).

For sure, the advent of imatinib has drastically changed the GIST natural history, with an overall manageable toxicity profile and only rare serious adverse events (7). This treatment has also questioned the standard criteria of treatment response assessment, only based on uni- or bidimensional changes in tumor size (8, 9). As a matter of fact, imatinib-induced tumor necrosis has led to put greater relevance to tumor density and metabolism in treatment response assessment to all tyrosine kinase inhibitors (TKIs), becoming a new paradigm for imaging in the era of precision oncology (10). In particular, positron emission tomography (PET) with 18-fluorodeoxyglucose (^18^FDG) has proven to be highly sensitive in the early assessment of tumor response, especially in cases where there is doubt or when the early prediction of the response could be clinically useful for patient management (11–15).

However, even if ^18^FDG-PET is generally thought to be more sensitive than morphologic imaging modalities for assessing early therapy response, several questions remain unanswered, including the appropriate time to monitor a therapeutic protocol, the PET-CT protocol used, and the therapy response evaluation criteria that should be used (16). Indeed, although the majority of studies report a general decrease in FDG tumor uptake after imatinib therapy, the time interval between baseline and follow-up FDG-PET studies varies significantly, ranging from 1 week after imatinib onset to several months after treatment (17, 18).

Herein, we described in detail a case of an exon 11 KIT mutated-metastatic GIST patient, who presented an unexpected metabolic progression at the early ^18^FDG-PET evaluation after 1 month of first-line imatinib, unconfirmed at the liver biopsy performed near after, which has conversely shown a complete pathological response. This report aims to understand the existence of this metabolic pseudoprogression in GIST at the beginning of imatinib therapy in order to avoid early treatment discontinuation.

## 2 Case presentation

In December 2020, a 78-year-old male patient, with a concomitant Philadelphia chromosome-positive chronic myeloproliferative neoplasm (MPN) treated with bosutinib, underwent duodenal resection and cholecystectomy, due to a duodenal GIST, diagnosed after an acute episode of melena and severe anemia.

**Figure 1:**
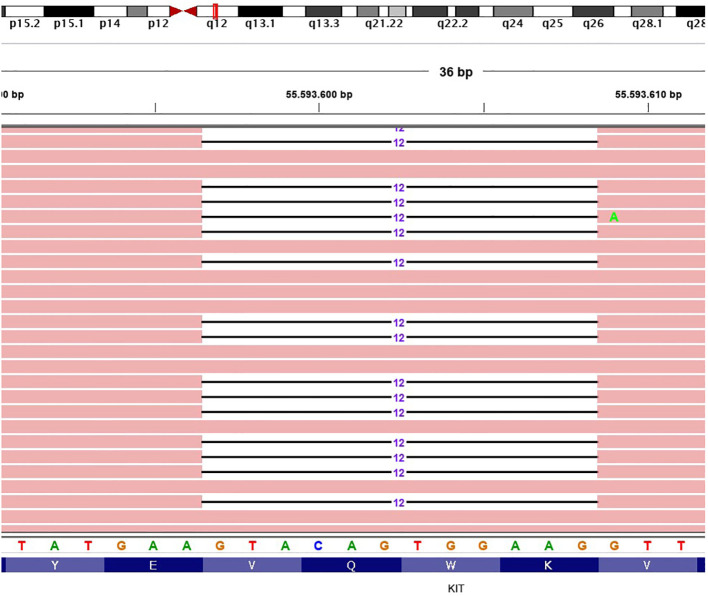
Representative graphical output of the next-generation sequencing analysis, showing an exon 11 deletion of KIT (Gln556_Val559del). The different colors are referred to the single DNA basis.

The histologic examination confirmed the diagnosis of a predominantly epithelioid cell GIST at high risk of relapse according to the Miettinen criteria [site: duodenum; size: 5.5 cm; mitotic index: 6/50 high power field (HPF)]. The microscopic margins were negative and there was no evidence of tumor rupture. The molecular analysis performed with next-generation sequencing (NGS) analysis showed an exon 11 deletion of KIT (Gln556_Val559del) (**Figure 1**).

**Figure 2:**
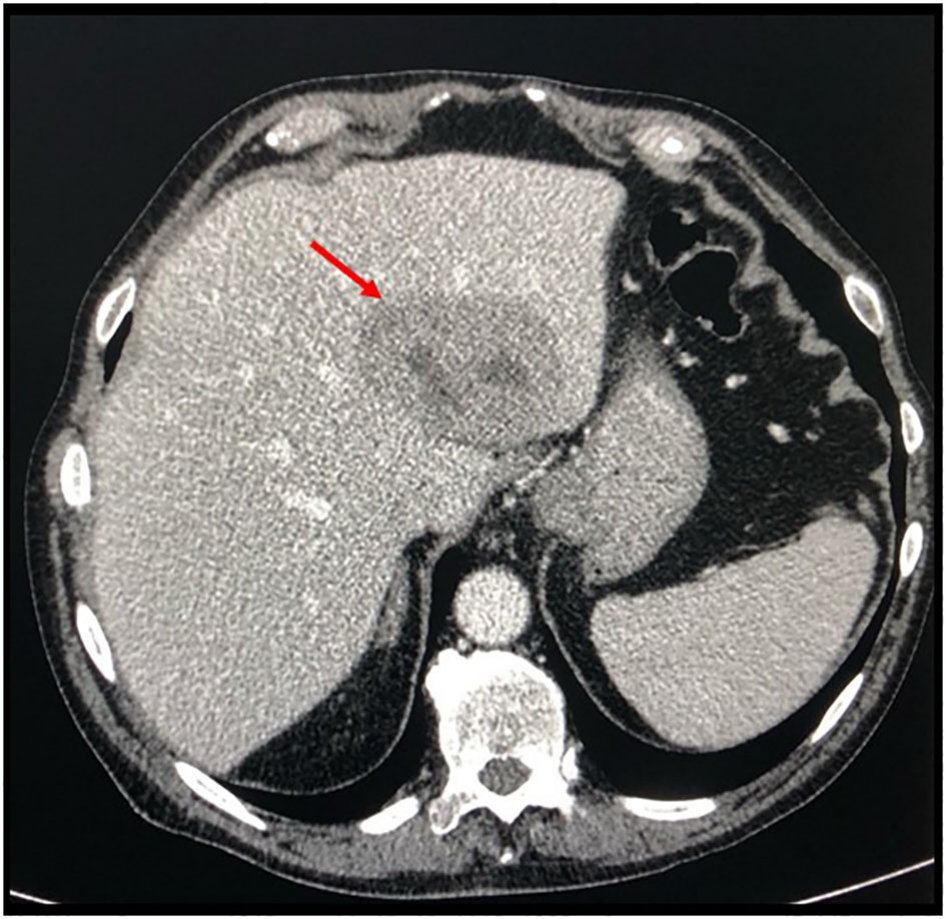
Basal axial CT scan evaluation, showing a hypodense and partially colliquated liver lesion at segments II–III (*arrow*).

**Figure 3:**
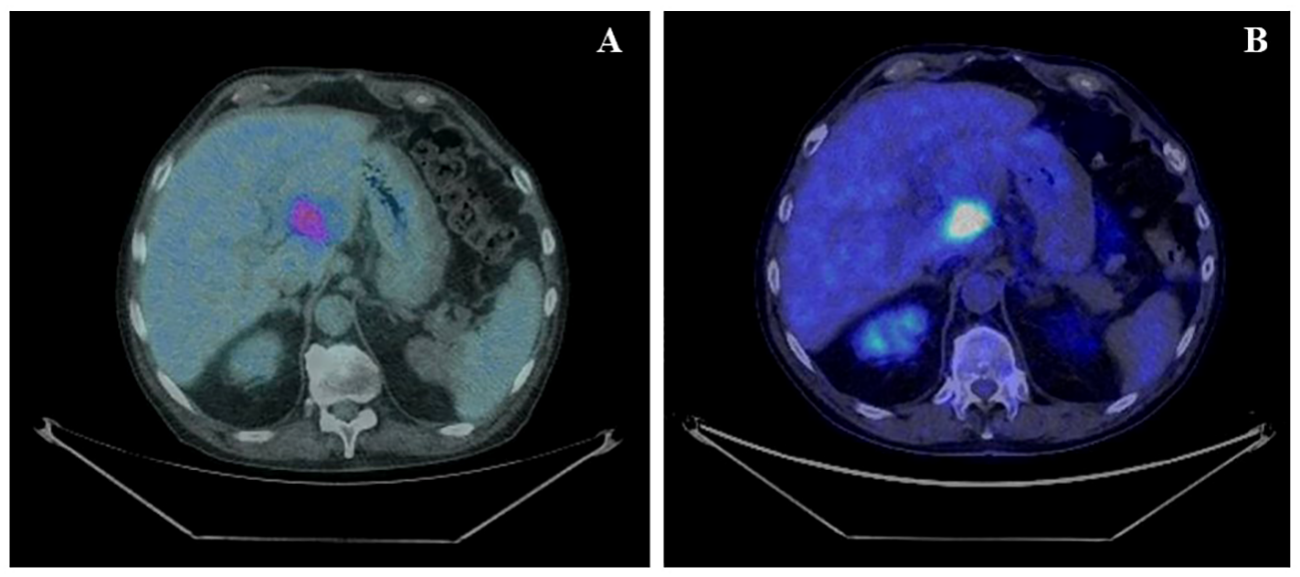
**(A)** Basal axial PET/CT fused images, showing a hypermetabolic lesion involving II–III liver segments (SUVmax 9.4). **(B)** Axial PET/CT fused images, restaging scan after a month of imatinib therapy: the hypermetabolic area appears increased in size and shows a greater ^18^F-FDG uptake: SUVmax 20.1 (vs. 9.4).

**Figure 4:**
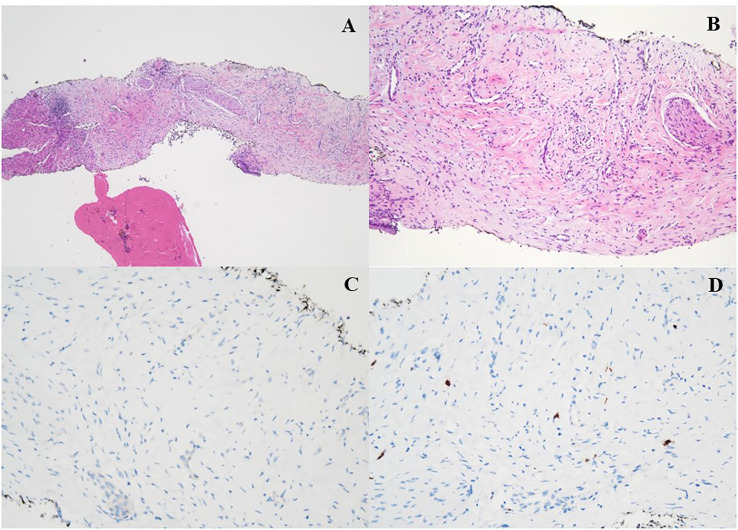
**(A, B)** H&E ×4: histologic features of the liver biopsy showing a vascular tissue with bland spindle cell fibroblast with collagenous stroma, admixed with a variable number of inflammatory elements (granulation tissue). **(C, D)** Immunohistochemical stain for DOG-1 **(C)** and CD117 **(D)** was all negative.

**Figure 5:**
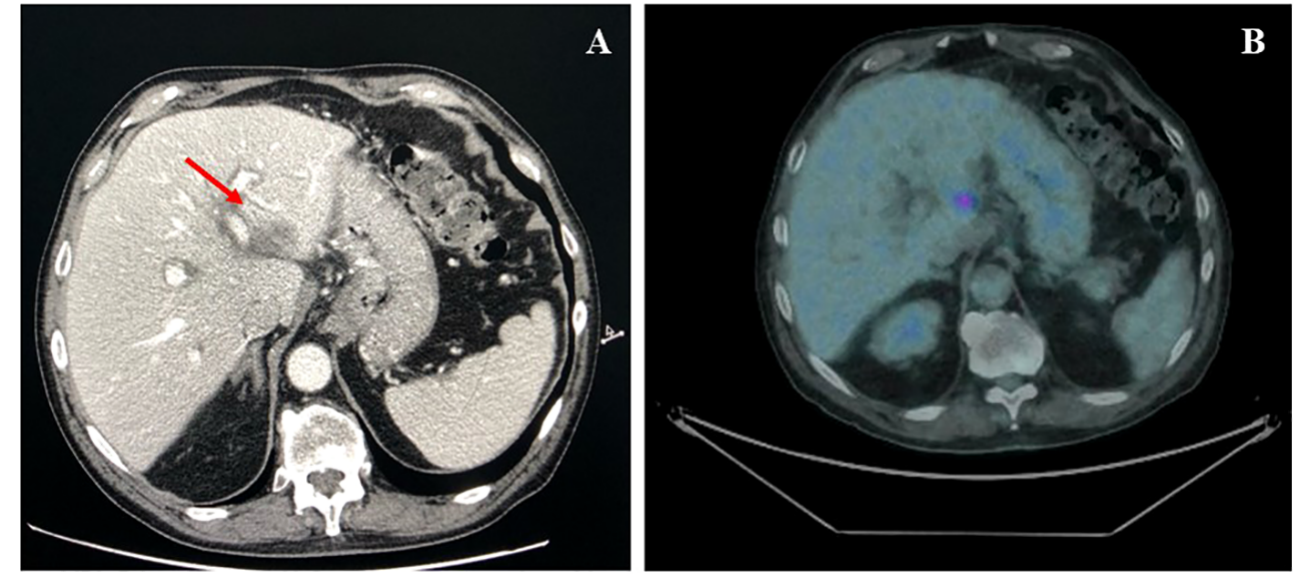
**(A)** Axial CT scan evaluation after 3 months of imatinib treatment (*arrow*), showing a partial response of liver lesion. **(B)** Axial PET/CT fused images scan, after 3 months of imatinib treatment, showing a marked decline in both lesion size and ^18^F-FDG uptake (SUVmax 7.9).

According to the risk class of tumor, an adjuvant treatment with imatinib 400 mg daily for 3 years should have been considered; however, after a multidisciplinary discussion together with hematologists, it was deemed more proper to continue treatment with bosutinib and reserve the therapy for GIST in case of disease relapse. During the surveillance program, a computer tomography (CT) scan performed in July 2022 showed a wide liver lesion at II–III hepatic segments with metastatic features (**Figure 2**). The subsequent ^18^FDG-PET/CT confirmed the presence of a hypermetabolic liver lesion with a maximum standardized uptake value (SUVmax) of 9.4 (**Figure 3**). According to the molecular profile, in August 2022, a first-line treatment with imatinib 400 mg daily was started. The subsequent ^18^FDG-PET/CT performed 1 month later for the early treatment response evaluation, given the concomitant chronic MPN already previously treated with imatinib, showed a metabolic progression of the liver lesion, presenting an SUVmax of 20.1 (**Figure 3**). Given this unexpected result, as compared with the exon 11 KIT-mutant molecular profile, a liver biopsy was performed. The histological examination has displayed liver tissue associated with paucicellular lesion of collagenized neovascularized fibrous–edematous stroma with lymphogranulocytic inflammatory infiltrate, such as granulation tissue (**Figure 4**). Based on this finding, suggesting a likely complete pathological response, imatinib therapy has been continued. The subsequent abdominal CT scan and ^18^FDG-PET/CT, performed in October 2022, 2 months after the beginning of therapy, revealed both a morphologic and metabolic partial response of the liver lesion, with an SUVmax of 7.9 (**Figures 5A, B**).

At present, the patient is still under treatment with imatinib at the standard dose, and at the last CT scan performed in July 2023, the liver lesion maintained the response, showing further dimensional reduction.

## 3 Discussion

The role of ^18^FDG-PET in the early assessment of tumor response in GIST has soon become a model for functional imaging of all other oncogene-addicted solid tumors treated with TKIs because it allows selecting those patients who can really benefit from molecular targeted therapies and conversely identifying those who are primarily resistant, even if the correlation between ^18^FDG-PET response and progression-free survival (PFS) is still controversial (12, 17). This is extremely relevant in doubtful cases or especially in those in which molecular profiling is lacking.

As widely known, kinase mutations have an undoubtful predictive value for sensitivity to imatinib, and the inclusion of KIT and PDGFRa mutational analysis in the diagnostic workup of all GISTs is now considered standard practice (6). Primary exon 11 KIT mutations, the most common ones in the KIT gene (67%), especially deletions, are known to confer the highest sensitivity to imatinib, and primary resistance can be considered a rare event (19).

In the presented case, the increase of FDG uptake after 1 month of imatinib was unexpected as compared with the molecular profile of the primary GIST. This has been the reason why tumor sampling of the metastatic lesion has been performed in order to confirm the GIST’s diagnosis of the hepatic lesion and exclude the presence of a resistant secondary mutation. The histological finding has conversely shown the presence of collagenous stroma strongly suggestive of a pathological complete response, and the surrounding abundant inflammatory infiltrate could explain the transient increased FDG uptake we found. Indeed, it is well established that activated inflammatory cells, especially those involved in inflammatory foci, show increased glucose metabolism, leading to a high FDG uptake (20). This evidence represents the pathophysiologic basis for the possible increase in FDG uptake in the case of a complete pathologic response, thus leading to false positivity on PET examination. This phenomenon referred to as the “flare effect” represents the underlying mechanism of pseudoprogression.

To our knowledge, this is the first case describing a metabolic pseudoprogression during the early assessment of tumor response in a metastatic KIT exon 11 mutant-GIST patient after 1 month of first-line imatinib. Even if extremely rare, clinicians should be aware of the possibility of this event, which should be interpreted according to the molecular profile if known, in order to avoid early treatment discontinuation. Therefore, an early metabolic progression during a molecular targeted therapy always deserves to be evaluated in the context of the disease molecular profiling, and in case of a discordant finding between functional imaging and molecular background, a short-term longitudinal control should be suggested.

Once again, GISTs have shown to be a model for functional imaging in the era of precision oncology, highlighting how all metabolic findings should be firstly interpreted together with the molecular data available.

## Data availability statement

The raw data supporting the conclusions of this article will be made available by the authors, without undue reservation.

## Ethics statement

Written informed consent was obtained from the individual(s) for the publication of any potentially identifiable images or data included in this article.

## Author contributions

ET: Conceptualization, Writing – original draft, Writing – review & editing. NC: Conceptualization, Writing – original draft, Writing – review & editing. GB: Data curation, Resources, Writing – original draft, Writing – review & editing. FP: Data curation, Resources, Software, Writing – review & editing. VS: Data curation, Resources, Software, Writing – review & editing. MGP: Data curation, Resources, Software, Writing – review & editing. DB: Writing – review & editing, Resources. MCN: Conceptualization, Writing – original draft, Writing – review & editing. MI: Data curation, Resources, Writing – review & editing. FC: Data curation, Resources, Supervision, Writing – review & editing. LL: Data curation, Resources, Supervision, Writing – review & editing. SF: Data curation, Resources, Software, Supervision, Writing – review & editing. MAP: Conceptualization, Data curation, Methodology, Supervision, Writing – review & editing. MN: Conceptualization, Writing – original draft, Writing – review & editing.
